# Supplementary material for: Are carbon emissions trading and green financial instruments synergistic? -Comprehensive quantitative research based on content analysis
Source: PLoS One. 2024 Mar 7;19(3):e0298601. doi: 10.1371/journal.pone.0298601 (PMC10919601; doi:10.1371/journal.pone.0298601)
Supplement: S1 Data — (ZIP) [file pone.0298601.s001.zip › Data/地级市Z_F工作报告.zip!/║┌┴·╜¡/╞δ╞δ╣■╢√╩╨/└┤╫╘╣1⁄2╓┌║┼í╛┬φ┐╦╩2╛▌═°í┐_2016.docx]

各位代表：

现在，我代表市人民政府，向大会报告工作，请予审议，并请各位政协委员提出意见。

一、“十二五”时期简要回顾及2015年工作总结

“十二五”时期，我们认真贯彻党中央、国务院和省委、省政府一系列方针政策，紧紧围绕市委的决策部署，全力以赴稳增长、调结构、促改革、惠民生，全市经济社会发展取得了显著成就。

五年来，坚持把发展作为第一要务，克服经济持续下行压力，抢抓国家“一带一路”、实施老工业基地振兴和我省建设两大平原现代农业综合配套改革试验区等一系列政策机遇，以发展装备制造、食品加工、新兴产业和现代服务业为重点，以项目建设为支撑，以改善发展环境为保障，促进了现代化大农业加快发展、工业经济实力不断增强和区域中心城市服务功能进一步提升，全市经济在困境中实现了稳步发展。全市地区生产总值年均增长7.9%，其中，一产、二产、三产增加值年均分别增长8.2%、7.1%和8.6%。公共财政预算收入年均增长9%。全社会固定资产投资年均增长10.8%。累计实施固定资产投资5000万元以上重点产业项目605个，完成投资1100亿元。社会消费品零售总额年均增长13.9%。

五年来，坚持以“三化”建设为引领，抢抓国家加快新型城镇化、加大基础设施投入的有利机遇，统筹搞好新城开发、老城改造和村镇建设，本着开发建设与生态保护并重的原则，着力推进道路、住房、绿化、“三供两治”等重点工程，使城市功能日趋完善、人居环境不断优化，成为国家新型城镇化试点城市、节能减排财政政策综合示范城市和生态文明先行示范区。全市建成公路总里程4090公里，升级改造市政道路115条。新建住宅小区219个、2200万平方米，城镇人均住房建筑面积达到29.2平方米。全市森林覆盖率和建成区绿化覆盖率分别达到10.2%和40.5%。城镇生活垃圾无害化处理率和污水集中处理率分别达到91.6%和74.5%。万元GDP综合能耗和主要污染物排放量均达到国家和省规定标准。

五年来，坚持民生为本，优先保障民生支出，统筹抓好各项社会事业发展，认真办好涉及群众利益的就业、就医、就学、社保、住房、养老、文体娱乐、社区建设、物业管理、安全生产、社会治安等民生实事，人民群众生活持续改善，幸福感和满意度进一步增强。全市城镇累计新增就业60万人次，登记失业率控制在4.1%以内。居民养老保险实现城乡一体化，城镇基本医疗、工伤、生育保险覆盖面逐步扩大，新农合参保率达到99%以上。城镇保障房建设和农村泥草（危）房改造总量位居全省前列。37.6万贫困人口实现脱贫。城镇、农村居民人均可支配收入年均分别增长11%和14.4%。

2015年，在市委的正确领导下，主动应对宏观经济形势带来的各种困难，坚持稳中求进，积极作为，全力保持经济平稳较快发展和社会和谐稳定，圆满完成了市十五届人大五次会议确定的重点任务。全市预计实现地区生产总值1270亿元，增长6.5%；全社会固定资产投资830.6亿元，增长10%；社会消费品零售总额683.5亿元，增长10.5%；城镇、农村居民人均可支配收入分别为23272元和12754元，增长9.3%和12.8%。公共财政预算收入完成79亿元，增长5.8%。

——项目建设扎实推进。重点推进固定资产投资5000万元以上产业项目269个，总投资514.6亿元，开复工率100%，完成投资224.2亿元，超额完成全年目标。20项省重点推进产业项目全部开复工，完成投资 30.1亿元。克服时间紧、任务重、困难大等因素，攻坚克难，敢打硬仗，按时完成了列入省督办的重点项目建设任务，齐富铁路增二线、滨洲铁路电气化改造工程征地拆迁和水利工程建设得到省政府充分肯定，棚改工作走在全省前列。北钢锻造产品优化升级等20个项目列入省“双百工程”。精心组织参加各类经贸交流招商活动，积极承接发达地区产业转移，引进5000万元以上招商项目191个，投资额168亿元。与中国铁科院、哈铁局及哈工大签署战略合作框架协议。全市各类园区累计完成基础设施建设投资89.5亿元，对项目的承载能力明显增强。

——现代农业加快发展。粮食产量稳定在300亿斤以上，实现“八连增”。落实“2513”高产创建工程核心区482万亩，增长19.3%。新增省级农民合作社规范社46个，入社耕地占集体耕地的80.5%。省级现代农机合作社规范社达到48个，占全省的41.1%，居全省首位。综合机械化程度达到95.1%。绿色有机食品基地面积达到1443万亩，销量和销售收入分别增长20.2%和25%。建立农产品生产全程监控物联网平台182个，成立了市级农产品检测机构，有机农产品质量安全溯源系统平台初步建成。全市农产品电商企业发展到188户，5户企业开办绿色食品营销网站。新建县乡村三级农业科技园区1567个。规模以上农产品加工企业发展到262户，农产品加工转化率42.5%。奶牛标准化规模养殖承载能力达到20万头以上，居全省首位。新增高档肉牛1万头，新建奶牛、肉牛规模养殖合作社78个。飞鹤乳业扩产增效，元盛、恒阳高端肉牛产业快速发展，嘉一香生猪养殖屠宰加工项目投产。建成全省首家动物无害化处理中心和西部地区动物疫病防控实验室。822个村完成土地确权外业实测工作。发放“四权”抵押贷款27.7亿元，增加4.5亿元。地方国有粮食购销企业改革稳步推进，引进合作资金30亿元，粮食仓容达到640万吨，是改革前的2.7倍。转移农村劳动力120万人，实现劳务收入136亿元。完成水利工程管理体制和产权制度改革试点任务16项。实施节水增粮118.9万亩，水土流失治理40万亩。建设农村饮水安全水源工程475处，近30万人饮水安全问题得到解决。争取财政专项资金2.4亿元，扶贫开发整村推进73个，脱贫人口11.6万人。

——工业经济平稳增长。规模以上工业实现增加值260亿元，增长3%，其中，装备制造业48.5亿元，下降18.7%；食品工业134亿元，增长19.5%，比重超过50%。县域工业增加值增长21.5%，高于全市18.5个百分点。完成老工业基地搬迁改造项目3个。黑化搬迁顺利启动。黑龙冰刀恢复生产。一重、北钢、齐轨道交通、昊华4户企业纳入直供电试点。向上争取企业技改资金1亿元。制定出台支持地方经济发展措施22条，为94户中小企业补办了产权证和土地证。新增规模以上工业企业45户。非公经济实现增加值671亿元，增长8.2%，占GDP的58.5%。积极开展科技型企业三年行动计划，新增一定规模科技型企业20户，新认定国家级高新技术企业7户，科技成果转化283项，高新技术产业增加值占GDP的11%。国企改革稳步推进，清理盘活3户国企闲置资产1.2亿元。厂办大集体改革作为全省试点在全国推广经验，完成744户企业认定与核资评估，移交“三供一业”项目13个，受益职工14.2万人。

——第三产业繁荣活跃。第三产业实现增加值567.8亿元，增长9.1%。为发挥财政资金杠杆撬动作用、有效吸纳社会资本，积极谋划建设北方畜产品交易中心，北方工业股权交易中心建设已获省政府批准。为给中小微企业创造更加优越的金融环境，创新开展了银税、银保合作和银企对接，设立了企业流动贷款周转金，为28户企业“倒贷”7.2亿元，为30个重点项目融资106亿元。安泰生物、正赫新材料、华工机床在新三板挂牌，瑞盛食品、千嘉木业、缘子汇福、蒲峪冰川苏打水在上海股权交易市场上市。实施“股权+债权”融资7.1亿元。全市金融机构各项存款余额1590.1亿元，贷款余额1407.7亿元，分别增加155.1亿元和243.3亿元。旅游业持续增长，全年接待游客1775万人次，实现旅游收入70.7亿元，分别增长15.5%和18.1%。奥悦室内外滑雪场及水上乐园、万力皮革美食城、昂昂溪水师森林温泉度假区开工建设，飞鹤乳业观光牧场、扎龙温泉小镇商业街、龙沙动植物园水上项目建成并对外开放。泰湖湿地公园和梅里斯哈拉新村分别晋升为国家4A和3A级旅游景区。罗西亚大街成为全国首批、全省唯一的中国历史文化街区，兴十四村、哈拉新村和小河东村被列为中国乡村旅游模范村。“互联网+绿色有机食品产业”发展迅速，线上交易额达43亿元。万达广场、翰沃家居建材城建成运营。对俄农产品贸易产业带项目正式启动。成功举办绿博会、小交会、农产品电商博览会等35个展会。与俄罗斯乌法市成为友好城市。

　　——城乡建设取得新成果。完成上一轮城市总体规划、小城镇规划和中心城区7个专项规划，城乡规划展示馆投入运行。哈齐高铁正式通车。卜奎互通和南互通工程交付使用。劳动湖南扩全线贯通。成功与中冶集团签订沿江景观带PPP项目合作协议。升级改造城区主次干道30条、铺装面积108万平方米，打通了市民期盼多年的双华路、明海公路、曙光北大街等城市主干道。投资1.2亿元，建设农村公路330公里。投资1.96亿元，建设“三供两治”项目10个。中心城区新建大热源3座，改造老旧供热、供水管网103公里，拆除供热小烟囱35座，撤并整合燃煤锅炉369台。氧化塘治理、污水处理等项目扎实推进。全市淘汰黄标车1.4万台，投放新能源公交车47台，亮化公交站点635处，新建公交首末站5处。城市管理重心下移，乡镇成立城管中队，县（市）区、乡镇城乡管理网格覆盖率分别达到98%和95%，7个县区建成数字化城管平台。中心城区拆除违建4830处，新建停车场22处，20座新型环保公厕投入使用。累计争取资金8亿元，推进72个节能减排项目实施，节能减排示范市综合考核全国优秀。12个单位晋升为国家级节约型公共机构示范单位。空气优良天数达313天。城区栽植树木、绿篱140.5万株，新增绿地面积128万平方米；乡村植树造林13.4万亩。新创建省级生态县4个，全市生态创建水平均达到省级以上标准。启动美丽乡村“三年行动计划”，在六条文明带上打造了191个达标村、166个典型示范村，走在全省前列。

——人民生活持续改善。30件民生实事完成29件，“冬季国际冰雪马拉松”赛事因合作方核心层人员变动无法实施。全市第一家创业咖啡成立运营，为9957人发放小额担保贷款5.3亿元。城镇新增就业10.4万人。城镇企业职工养老保险参保52万人，医疗、工伤、生育保险参保分别达到174.3万人、38.8万人和18.3万人。实行医保城乡统筹，完善城乡居民医保大病制度，启动和推进医保市级统筹工作，群众就医负担进一步减轻。城乡低保标准分别提高到每人每月510元和每人每年3500元，集中供养“三无”人员生活补贴实现年度动态提高，临时救助困难家庭1.3万户。养老机构老年人意外伤害保险和医疗签约服务逐步推行，智慧社区居家养老服务体系试点取得成效，失能老人康复中心养老护理实训基地和民康医院项目主体完工，中医医院百草养生院投入使用。困难群体法律援助实现全覆盖。实施精神障碍人员救助和残疾人关爱工程，“健康快车”免费为1280名白内障患者进行复明手术。新开工建设棚改回迁房2.2万套，建成7901套，启动49个地段3.1万户棚改征收任务，购买1.79万套存量商品房用于棚改回迁；建设公租房1530套、9.2万平方米；实施既有建筑节能改造261.3万平方米，其中中心城区探索合同能源管理方式改造12万平方米；完成城区老旧单散住宅小区改造123万平方米，受益居民2.4万户；改造农村泥草（危）房3.2万户，超额完成计划任务。取消了两座嫩江大桥收费。下调了城区供热取暖费价格，规范了中心城区机动车停放服务收费行为。新改扩建幼儿园46所、标准化学校21所，义务教育均衡化稳步推进，我市成为全省唯一的“省部共建国家现代农村职业教育改革试验区”试点市，3个县成为全国农村职成教育示范县。县级公立医院改革全面铺开。成功举办国际鹤文化艺术节、扎龙国际婚礼文化节、中国老年合唱节、全民健身运动会等重大群文活动。3部影视作品荣获国家级大奖，神鹤剧本工厂吸引投资1.2亿元。满绣入选国家非物质文化遗产名录。新建中心村文化设施107处，新增全民健身场地300处，承办亚洲冰球联赛等国家级以上赛事6项，市射击综合训练馆建设完成。城乡社区“双百”示范工程扎实推进，新建城市、农村示范社区21个，我市成为全省首批社区社会工作试点市。全国双拥模范城实现“七连冠”，全国“六五”普法通过验收。深化“平安鹤城”、“法治鹤城”建设，刑事案件下降6.8%，荣获全国法治城市创建先进单位。严厉打击各种扰乱市场经济秩序行为，连续三年被评为“中国十佳食品安全城市”。信访形势总体稳定。安全生产责任有效落实，专项整治成效显著，呈现稳中向好态势。

——政府职能进一步转变。扎实开展“三严三实”专题教育，严肃整治“不严不实”问题，政府服务效能、创业劲头和担当意识进一步增强。推进简政放权，市本级行政权力取消125项、暂停10项、下放10项，承接省下放权力92项。全面取消市级非行政许可审批类别，编制并公开了权力清单、责任清单和权力运行流程图，开展第三方评估，主动接受监督。实施“三集中、三到位”改革，建成市、县（区）、乡、村四级政务服务体系。优化审批流程，集中审批事项全部实现一口受理、一门办结，网上行政审批中心实现省、市、县三级互联互通，重点投资项目实行全程代办服务。增强市区食品药品、城市管理、环境监察执法力量，规范细化行政执法自由裁量权基准1179项。公益类和经营类事业单位类别批复工作全面开展。非应急类便民服务热线实现“‘12345’一号通”，群众诉求办结率达97.9%。全年查处破坏经济发展环境案件104件。统一规范中介机构服务收费、时限和行为，全面推行四证合一、一照一码、先照后证等工商注册便利化制度改革。出台市政府重大行政决策与执行实施规范，完善政府法律顾问制度。深入贯彻《预算法》，发挥监察、审计、绩效评价作用，强化预算刚性执行，全市“三公”经费支出下降8.3%。监督审查政府投资项目318个，节约投资10.4亿元。

民族宗教、新闻出版、社会科学、妇女儿童、防灾减灾、地方志、统计、档案、人防、气象等各项工作，也都取得了新成绩。

2015年，我们在做好各项工作的同时，主动适应经济发展新常态，通过对上级政策的深入研究和对市情实际的深刻把握，努力找准政策机遇与我市发展的契合点，进一步明确了新常态下的产业发展方向和路径。依托存量扩张和溢出、资源深加工、创新驱动、招商引资、加强与资本市场合作、强化与科研单位联姻，以市场化意识和专业化操作，系统谋划装备制造、食品加工、特色轻工以及旅游、文化、体育等产业并大力组织实施。在装备制造业上，立足工业基础，重点发展整机装备制造、基础零部件、金属新材料、节能环保等产业；在食品加工业上，立足资源优势，重点发展绿色有机食品、马铃薯、肉类加工、乳制品等产业；在特色轻工业上，立足市场需求，重点发展工艺陶瓷、冰雪运动器材、白酒、高端饮用水等产业，为实施“十三五”规划开好局、起好步，重振我市经济雄风夯实了基础。

各位代表！2015年全市经济社会发展取得的成绩，是省委、省政府和市委正确领导的结果，是历届市政府奠定良好工作基础的结果，更是全市上下团结一心、共同努力的结果。在此，我代表市人民政府，向全市人民，向驻齐解放军、武警官兵和中省直单位，向所有关心、支持齐齐哈尔发展的各界人士，表示衷心的感谢！

在看到成绩的同时，我们也清醒认识到工作中存在的问题。主要是：传统装备制造业受外部形势影响较大，对全市工业和整体经济当期呈现负向拉动，产业发展中体制机制不活和市场化、专业化程度不高等深层次矛盾仍然存在；农业结构调整任务艰巨，各县（市）区主导产业仍不突出；牵动发展的大项目、好项目不多，存量扩张、资源精深加工不够，若干产业领域的增长潜力还没有充分释放；创新能力不强，在新常态下依靠新动能推动经济发展的动力不足；财政收支矛盾突出，人均财力水平低，民生事业和城市建设历史欠账多，保“吃饭”、保运转面临很大压力；一些行政行为与“三严三实”要求还有很大差距，干事创业的精气神还有待于进一步提升，发展环境需要持续大力改善。对此，我们一定坚持问题导向，高度重视，找准对策，认真解决。

二、“十三五”时期规划目标及2016年工作任务

“十三五”时期是全面建成小康社会的决胜阶段。在新常态的大背景下，国家宏观经济处于换挡期，新的发展动能逐渐发力，新模式、新技术、新业态不断涌现，我们正处在艰难的适应期和调整期。“不适应则退、慢适应也退”。先行一步，我们就能适应新常态，实现新发展；落后半拍，我们就会失去机会，贻误发展。我们将在相当长的时期内面临着转型发展带来的巨大压力和严峻考验。但从总体上判断，我市发展趋势是向好的，机遇大于挑战。国家实施新一轮老工业基地调整改造和“中国制造2025”战略，为我市装备制造业转型升级提供了强力支撑；我市作为国家“一带一路”特别是“龙江丝路带”的重要节点，具备良好的区位和交通优势，拥有通往欧洲较大规模的昂昂溪三间房铁路货车编组站，对外开放和经贸合作将迎来利好机遇，把握住这一机遇，我市就能更好地利用国际国内两个市场、两种资源，就能从“边缘城市”变成“前沿城市”；2022年北京冬奥会的举办，对我市具有比较优势的冰雪体育运动、训练、器材生产以及由此衍生的关联产业发展将带来更多商机；随着改革的全面深化以及“互联网+”行动计划和“大众创业、万众创新”政策的实施，改革、转型和创新逐步成为我市经济发展的新动力；国家加大精准扶贫和基础设施建设投入力度，将为我市贫困县整体脱贫和城市改造带来政策机遇；国内市场对健康性、安全性食品和生态环境的需求日益迫切，我市凭借优质的自然生态和高品质的绿色食品，在绿色有机食品产业以及旅游业、养老服务业等领域蕴藏巨大发展潜力；我市拥有从材料、关键部件到整机制造完整的装备制造产业体系，既能立足基础推进存量扩张和技术溢出，又能通过创新驱动和招商引资，在高端装备制造、金属新材料、节能环保等领域实现新突破。只要我们紧紧抓住这些宝贵的发展机遇，坚定信心，凝聚力量，千方百计破解发展难题，扎扎实实推进潜力释放，鹤城大地就一定能够呈现出新的发展之势、振兴之势、崛起之势。

“十三五”时期，我们要深入贯彻党的十八大和十八届三中、四中、五中全会以及省委十一届六次全会精神，坚持“四个全面”战略布局，牢固树立“五大发展理念”，主动适应经济发展新常态，着力推进结构性改革，加快培育新的发展动能，按照市委十二届八次全会总体部署，以创新为主线，以项目为支撑，以增进城乡居民幸福感和获得感为目标，坚持市场化意识和专业化操作，大力发展装备制造、食品加工、特色轻工以及旅游、文化、体育、养老服务等具有供给竞争优势的产业，统筹推进经济、政治、文化、社会和生态文明建设，与全省全国同步全面建成小康社会。到2020年，全市地区生产总值年均增长6.5%以上，比2010年翻一番；城乡居民人均可支配收入提前两年翻一番；现行标准下全市31.6万贫困人口提前一年全部脱贫；8个国家级和省级贫困县全部摘帽；力争有1个县进入全省十强、3个县进入全省前二十名。届时，我市产业实力更加雄厚，城乡发展更加协调，生态环境更加优良，对外开放更加深入，发展成果更加普惠，人民生活更加幸福。

“十三五”时期，我们在发展中要牢牢把握好以下五点：

一是坚持创新发展。依靠供给侧结构性改革、科技创新和人才，在装备制造、食品加工、特色轻工、现代服务业等具有供给竞争优势的领域，孕育新的发展动能，形成整机装备制造、基础零部件、金属新材料、节能环保、绿色有机食品、工艺陶瓷等一批新的产业群、产品群，努力把齐齐哈尔打造成创新引领、动能强劲、人气集聚的活力城市。

二是统筹协调发展。注重城乡一体、产城一体，加快新型城镇化、产业园区和村镇建设；注重县域协作、优势互补，大力发展县域经济，坚决打赢脱贫攻坚战；注重产业之间、经济和社会之间协调发展，努力把齐齐哈尔打造成融合发展型城市。

三是实施绿色发展。充分利用北纬47度特殊区位，巩固提升全市整体生态优势，推进节能减排示范市和美丽乡村建设，加强资源节约和环境保护，努力把齐齐哈尔打造成国内著名的生态示范城市。

四是加快开放发展。发挥我市作为“一带一路”特别是“龙江丝路带”重要节点和区域中心城市的优势，以辐射周边为基础，进一步密切与国内发达地区交流合作，并逐步向俄欧、日韩以及港澳台地区拓展，努力把齐齐哈尔打造成全方位开放城市。

五是实现共享发展。坚持积极作为、量力而行，守住底线，补齐短板，注重机会公平、服务均等，保障基本民生，共享改革和发展成果，让全市人民共同迈入全面小康社会。

2016年是全面建成小康社会决胜阶段的开局之年，也是推进结构性改革的攻坚之年。我们要按照中央经济工作、城市工作、农村工作会议和省委经济工作、扶贫开发工作会议要求，稳增长、调结构、惠民生、防风险，精准发力，扎实起步，努力实现“十三五”良好开局。主要预期目标是，全市地区生产总值增长6.5%左右，公共财政预算收入增长2.3%，城镇、农村居民人均可支配收入分别增长8%和9%以上，城镇登记失业率控制在4.5%以内。

实现上述目标，关键是围绕产业上项目、围绕发展抓改革、围绕民生办实事。重点做好以下工作：

（一）全力推进项目建设。紧紧抓住项目建设不放松，通过存量扩张、资源深加工、创新驱动、招商引资等路径，全力打好项目建设攻坚战。建立倒逼机制，强化督办问责，按时完成列入省督办的重点项目。以装备制造、食品加工、特色轻工以及旅游、文化、养老服务等产业为主攻方向，重点推进固定资产投资超5000万元产业项目260个以上。围绕存量资产实施技术升级、合资合作，通过盘活存量做大增量。依靠农业全产业链上项目，深度开发利用“原字号”资源，加快把资源优势转化为经济优势。促进科技成果转化落地，生成一批金属新材料、节能环保等新兴产业项目。创新招商方式，大力开展定向招商、以商招商和专人招商，加快引进一批具有国际水准、代表产业高端的重大项目。采取市场化手段，加快园区基础设施建设，增强园区对项目的承载力。全社会固定资产投资增长8%左右。

（二）加快调整农业结构。结合两大平原现代农业综合配套改革，以10个农村重点产业和项目为牵动，以稳产能、调结构、提品质、增效益为目标，促进粮经饲统筹、农林牧渔结合、种养加一体、一二三产业融合，加快发展现代化大农业。根据各地资源禀赋、产业基础和市场需求，优化种植业结构。大力压缩玉米种植面积；稳定大豆面积，提升大豆品质，发展专用豆；扩大水稻、马铃薯、杂粮、饲草饲料种植面积，分别增加50万亩、30万亩、100万亩和100万亩，尤其是依托马铃薯繁育优势，积极扩大种薯生产规模，建设克山、讷河种薯生产基地；引导推广瓜果蔬菜、中草药、食用菌、花卉等特色种植。扶持企业自建高标准绿色基地230万亩、有机基地3万亩。积极打造农业全产业链发展模式，加快龙头企业整合及产业联盟组建，绿色、有机食品加工龙头企业发展到60户。制定绿色有机食品生产标准，加强检验检测，做实绿色品牌。加快“互联网+农业”发展，搞好电子商务平台建设，确保“既要种得好、更要销得好”。深入实施“两牛一猪”发展规划，加快雪牛扩繁改良，新建和改扩建300头以上标准化奶牛、肉牛养殖场80个，年出栏3000头以上标准化生猪养殖场30个。积极推进奶山羊规模养殖。加强农业社会化服务体系建设，提高合作社发展质量，扩大省级规范社比例，鼓励合作社由生产主体向全方位经营主体转变。加快推进农村土地确权工作，累计完成外业实测村数达80%以上。深化农村金融改革，鼓励各县（市）合作组建融资担保平台，涉农贷款投放占全部贷款50%以上，“四权”抵押及活体抵押贷款总量增长10%以上。完善农业保险体系，健全新型农业经营主体风险缓释和补偿机制，降低农业生产风险。持续推进地方国有粮食购销企业改革，确保35户参改企业全部改革到位，新增仓储能力100万吨。适时启动国有林场和供销社体制改革。以54处万亩灌区、140处大中小型水库工程为重点，全面推进水利改革。新增节水灌溉面积10万亩，水土流失治理面积10万亩。

（三）依靠供给竞争优势带动工业经济加速发展。围绕落实“中国制造2025”规划，推进高端制造和“互联网+工业”，增加有质量、有效益的产品供给。规模以上工业增加值增长4.5%。在整机装备制造产业上，抢抓国家加快高铁建设和支持新能源汽车发展机遇，进一步释放齐轨道交通、精铸良、龙华汽车等企业产能，大力发展铁路装备制造产业、新能源汽车和房车；深化与中国钢铁研究总院、航天五院、中冶集团、中国农科院等科研院所合作，加快发展核电、环焊机、数控金属切削及各类高端专用机械、成套冶金设备、马铃薯主食化食品加工机械制造等项目。在基础零部件产业上，推进与中国空军装备研究院、中船重工703所、哈工大、哈工程等合作，加快燃气轮机零部件、力矩电机、雷达极化装置等项目建设；引进战略投资者，加快腾翔铸锻造启动生产，补齐铸造短板。在金属新材料产业上，依托北满特钢，加快金属新材料产业园区建设；抓好铜冶炼项目，建设铜产品加工园；推进与北京华钢联、香港嘉瑞等合作，使CSU球团、新型复合板材、铝稀土中间合金等重点项目尽快落地投产；加快纳米增强车轴钢、锰钼高强度合金钢及轨道加工项目科技成果产业化；依托军工优势推进军民融合项目，打造碳纤维高性能复合材料基地。在节能环保产业上，推进秸秆综合利用、微生物菌床、寒地供暖保暖、直驱力矩节能电机和相变材料产业化深入实施。在食品产业上，围绕玉米、水稻、马铃薯、水、乳、肉等资源的全产业链，加快飞鹤奶山羊乳制品、瑞盛马铃薯主粮化、淼源大豆功能乳饮料等食品加工项目建设，支持安泰、元盛、恒阳等具有竞争优势的食品企业做大做强。同时，通过对外合作，在艺术化生活陶瓷和冰雪运动器材产业上加快破题，并逐步发展壮大。认真落实国家和省、市扶持中小企业发展政策，支持中小微企业发展壮大。主动对接国家600亿元中小企业发展基金，实施非公经济发展三年行动计划和中小企业成长工程，使一批成长性好的中小企业进入规模以上行列。全力支持一重与哈电合并重组。稳步推进国企改革和厂办大集体改革，进一步释放企业发展活力。

（四）大力发展现代服务业。围绕我市特有的生态文化、冰雪运动、健康养老等供给优势，推进现代服务业快速发展。突出以“鹤”为主题的旅游品牌，大力发展旅游业。集中打造齐扎、滨江两条旅游观光带和碾子山旅游小镇，重点推进碾子山奥悦滑雪场、龙沙奥悦室内滑雪场及水上乐园、梅里斯民族文化旅游、昂昂溪水师森林温泉度假区和格林水世界等项目。建设好省第二梯队冬季旅游线路节点。加强旅游营销，着力开发具有地方特色的旅游产品和纪念品。启动“舌尖上的鹤城”活动，规划建设特色美食街。大力发展文化创意产业，推进文化与旅游、体育等多产业相融合，做大做强工艺陶瓷、嫩江玛瑙、满绣等本地特色文化产品，推出一批有代表性的演艺精品。抢抓2022年北京冬奥会机遇，做足冰雪运动文章，创建冰雪重大赛事活动基地。坚持医养结合，支持养老服务业发展，打造全国候鸟式养老基地。支持中汇城、万达广场、万力皮革城等建成项目发挥效益，加快培育建华星光蔬菜、龙江农鲜达、富拉尔基华为科技等一批具有竞争优势的电商企业。社会消费品零售总额增长12%以上。强化金融支撑，优化信贷结构。深化银税、银保合作，建立担保体系和风险补偿机制。设立工业、农业和节能减排三支产业发展引导基金。推进北方工业股权、北方畜产品和东北亚北方商品交易中心建设，继续鼓励扶持企业上市融资。积极争取在我市设立更多的金融分支机构。

（五）以壮大县域经济带动整体脱贫。把抓产业、建园区、上项目、增税源作为强县富民的根本之举，做大做强支柱产业，抓好抓实特色产业，形成“一县一品、一品多县、县县有主导产业”的发展格局，带动县域经济赶超晋位和贫困县整体脱贫。按照差异化、特色化方向，依托资源禀赋和比较优势，推进高档肉食、主食业、薯业、酒业、乳业、杂粮、陶瓷及高端饮用水等立县产业发展。整合资源，优化配置，鼓励县域之间、企业之间跨地域、跨行业协作发展，建立特色产业集群，提升市场竞争力。编制县域特色优势产业招商指南，积极寻求合作线索，努力生成新的项目。加强重点产业和项目督办考核，促进达产达效。强化县域商贸物流、电子商务平台体系建设。全面推进精准扶贫、精准脱贫，因地制宜、因村施策、因户扶持，坚决完成2016年节点脱贫任务，减少贫困人口12万人。实施“开发式”脱贫，大力发展特色产业，引领群众增加收入；实施“技能式”脱贫，开展职业技能培训，鼓励扶持贫困群众创业就业；实施“扶志式”脱贫，以结对帮扶、教育引导为主，激发贫困群众脱贫的内生动力；实施“兜底式”脱贫，对丧失劳动能力的贫困群体给予兜底性保障政策，将符合政策的贫困人口纳入低保范围。大力支持革命老区、少数民族地区脱贫攻坚，支持梅里斯创建国家民族团结进步示范区。

（六）进一步扩大对外开放。完成以昂昂溪三间房为重点的全市物流业发展规划，并适时组织实施。积极搭建对俄、对欧互通平台，引进大型仓储物流及加工企业，打造外向型产业集聚区和对外商品集散地。抓好对哈长城市群发展规划的跟踪与衔接，积极申请建设国家级产业合作发展与转型升级示范区，拓展区域经济发展空间。推进梅里斯对俄农产品贸易，加快建设外向型产业园区。支持跨境贸易电商等新型业态发展。实施机场改造工程，争取开通新航线。支持铁路电气化改造，加密哈齐高铁车次。建设江桥黑蒙、嫩通高速至嫩江大桥、牙克石至四平碾子山段公路，完善区域交通网络，实现空铁公联运一体化。高质量办好绿博会、小交会、国际鹤文化艺术节等重要会节。加强与欧洲高端装备制造区、冰雪运动发达国家交流合作，促进与国内外友好城市经贸文化往来。

（七）统筹抓好城乡规划、建设和治理。发挥规划引领作用，探索推进国民经济、土地利用、城市规划“多规合一”，启动新一轮城市总体规划修编，做好城市地下综合管廊等一批专项规划。抓住新型城镇化试点机遇，既最大限度争取国家政策支持，又注重采取PPP、资产证券化等模式，积极吸纳社会资本，多元筹措建设资金，及时启动我市城市基础设施建设三年计划。以中心城区环路建设为重点，启动通北、腾飞东等西北、东北、东南环路建设和齐齐哈尔站至高铁南站联接线前期工作。新建和改造城市污雨水管网及泵站，实施污水截流、雨水排放畅通工程。完成和平热源建设并投入使用，改造老旧供热管线40公里，同步推动卜奎、腾飞两大热源建设。强化供热管理，确保供热质量和安全。推进生活垃圾发电、餐厨垃圾和污泥协同处置等项目加快实施。做好城市地下综合管廊建设前期准备工作，积极争取成为国家试点、示范城市。以棚户区改造为重点，推动房地产业健康发展。完成劳动湖南扩沿岸广场、桥梁、堤顶路及岸线亮化工程建设。完善扎龙温泉小镇基础设施配套建设。抓好龙江县国家级基础设施投融资体制改革试点。推进城管综合执法和环卫市场化运营，开展城区环境综合整治，将南苑新城、北苑凤凰城等新开发区域纳入城区日常保洁。大力推进生态文明建设，加快建设节能减排示范市和生态文明先行示范区，创建省级生态市，启动高新区低碳城市生态试点。万元GDP综合能耗下降5.9%。控制主要污染物减排，化学需氧量、二氧化硫、氨氮、氮氧化物分别削减2.29%、4.44%、4.46%和3.56%。加强湿地保护管理。植树造林7.2万亩。深化美丽乡村建设，统筹抓好硬件设施提标和村风民风转变，集中打造一批典型示范村和达标村。

（八）强力推动“大众创业、万众创新”。构建以众创、众包、众扶、众筹为核心的“大众创业、万众创新”支撑平台，大力发展基于“互联网+”业态下的创业活动，建设一批小微企业创业创新基地，降低全社会创业门槛。发挥人才在创新创业中的引领作用，在全社会营造尊重人才、培养人才、引进人才、留住人才的良好环境和浓厚氛围。设立“两创”助保贷款，深化商事制度改革，强化创业政策扶持，鼓励在外大学生、农民工返乡创业，引导高校和科研院所科技人员依靠市场化组织方式创新创业。建立各类众创空间20家以上,城镇新增就业8.6万人。深化高新区体制改革，加强项目孵化和引进，加快高新技术产业发展。高新技术产业增加值占GDP比重达到11%以上，科技成果转化290项以上。继续推进科技型企业三年行动计划，新增一定规模科技型企业31户。抓好商标战略示范城市创建，扩大品牌影响力。

（九）着力保障和改善民生。在财力使用上，坚持民生优先。继续扩大社保覆盖面，推进省内异地就医即时结算。促进教育公平和义务教育均衡发展，确保克东、富裕、铁锋、龙沙、建华5个县区通过均衡发展验收；切实加强素质教育，全面提高教育教学质量，推行艺体特色教育“一条龙”后备人才培养模式；面向市场办学，优化职业教育专业设置，增加工艺陶瓷、冰雪运动训练等专业；建立高校与地方合作机制，让本地高校人才和技术更好服务地方经济。推动大医联体建设，启动城市公立医院改革，加快市第一医院南院使用运营。积极做好“全面二孩”政策宣传引导工作。加强基层公共文化服务网络建设，开展好重大群众文化活动。建立养老机构评价指标体系，实现规范化管理、人性化服务。加强物业管理，提升业主满意度。争创4个国家级、5个省级综合减灾示范社区。深入开展法治宣传教育，完善立体化社会治安防控体系，严厉打击各类扰乱市场秩序和各种严重危害公共安全的违法犯罪活动。加强价格监管，保障消费者权益。集中化解重点信访事项。深入推进安全责任落实，强化生产、消防、交通、特种设备等领域安全监管和隐患整治。加强基层食品药品监督管理机构标准化建设，完善食品药品监管体系。

重点抓好25件民生实事。（1）改造棚户区2万户，建设公共租赁住房800套。（2）综合改造低温楼200万平方米。（3）启动中心城区新源净水厂建设。（4）改造站前北大街、黎明街、南荟街等影响群众出行的“卡脖路”、“破损路”8 条。（5）实施“结构危楼”改造工程，解决影响群众居住安全问题。（6）实施城区老旧单散小区管网综合改造工程，解决居民供暖和给排水问题。（7）实施工人文化宫区域综合改造工程。（8）修缮清真寺。（9）推进污水提标改造和氧化塘综合改造工程，排放标准由一级B提升至一级A。（10）实施农村饮水巩固提升工程，新增受益人口5万人。（11）拆除违建3000处以上。（12）市区新建临街停车场30个。（13）新改扩建道路植树（绿篱）104万株、新建绿地20公顷，完善鹤城公园和南站广场绿化工程，修缮龙沙公园部分甬道。（14）改善环卫工人作业条件，市本级财政供养环卫工人月工资待遇提高到1400元。（15）更新清洁能源和新能源公交车辆80台，增加高铁南站公交车次；投放出租汽车750台。（16）淘汰老旧机动车1.2万辆，清理居民小区和临街“僵尸车”。（17）建成15个500平方米以上的农村示范社区。（18）建设义务教育标准化学校25所。（19）就业技能培训、创业培训和农村劳动力输出培训5万人，发放小额担保贷款2亿元。（20）浇筑室外冰场160块，新增健身器材120套;市区扩展沿江健身步道至10公里，打造5处体育主题公园。（21）建设鹤城烧烤和嫩江鱼生态农庄美食街。（22）深入实施城乡医保一体化改革，报销上限提高到15万元。（23）成立齐齐哈尔市老年协会，建设齐齐哈尔市老年大学。（24）新增“三无”精神障碍人员床位1000张；对有暴力倾向的精神障碍人员实行集中治疗供养。（25）启动贫困残疾人关爱行动，为160名贫困残疾儿童提供康复医疗和训练救助，为1000名贫困残疾人免费配发助视器、助行器、助听器，对市区400户贫困残疾人家庭进行无障碍改造，对市区150名升入高等院校的特困残疾人考生和贫困残疾人家庭子女给予一次性助学补贴。

（十）切实加强政府自身建设。巩固“三严三实”专题教育成果，真正以“严”的精神和“实”的作风，肩负起新常态下的发展重任。坚持依法行政，健全政府法律顾问制度，进一步规范行政行为，切实加强法治政府建设。持续推进简政放权、放管结合，完善规范权力清单和责任清单，最大限度向市场、社会和基层放权。完善网上审批，减少审批环节，实行“一口受理”。整合公共资源交易中心，建立统一规范的电子交易系统。深化行政执法体制改革，清理规范涉企行政事业性收费，坚决查处破坏发展环境的人和事。主动接受人大法律监督和工作监督、政协民主监督、媒体舆论监督和群众社会监督，认真办理人大代表建议和政协委员提案。严格执行《预算法》，全面实行零基预算，增强预算执行的刚性约束。将所有列入预算的公共资金、重大投资和重点工程全程纳入监察、审计监督，保证公共资金安全高效使用。牢固树立过紧日子思想，厉行勤俭节约，严控“三公”经费。在政府系统严格落实党风廉政建设责任制，坚持“一岗双责”，强化主体责任。加强对新业态、新技术和新生事物的学习与运用，不断提高专业素养，积极认识、适应、引领经济发展新常态，加快转换发展动能。努力争取国家和省密集出台的、含金量较高的重大利好政策，切实把政策用足用好、落实到位。在产业发展、项目招商、企业上市、规划建设管理等方面，注重采取市场化手段和专业化操作，不断创新工作方式方法。对生态保护、民生保障、脱贫攻坚等项工作，实行最严格的考核问责，确保把全部精力都用到工作落实上。

各位代表！过去的成绩令人鼓舞，美好的蓝图催人奋进。让我们在市委的正确领导下，万众一心，奋勇拼搏，为夺取全面建成小康社会决胜阶段的伟大胜利、创造齐齐哈尔更加美好的明天而努力奋斗！
